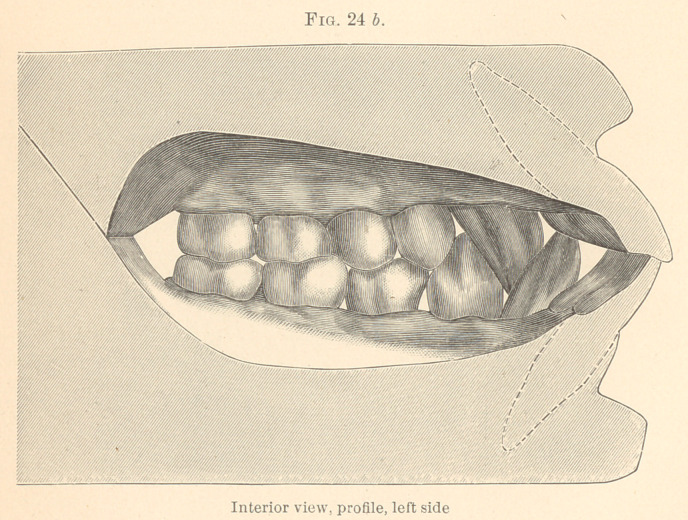# Articulation of the Teeth

**Published:** 1892-01

**Authors:** Isaac B. Davenport

**Affiliations:** Paris, France


### International Dental Journal.

Vol. XIII. January, 1892. No. 1.

### Original Communications.[1]

### ARTICULATION OF THE TEETH.[2]

BY ISAAC B. DAVENPORT, M.D., M.D.S., PARIS, FRANCE.

The old man who had lost all but two of his teeth and thanked God that those met, had some conception of the value of articulation.

Many people with the mouth half full of teeth could not thank God for more masticating surface than this old man had, because their teeth strike together so badly that only a few are capable of mastication. The fact that such are often pretty healthy people does not in the least prove that a very limited masticating surface is not most deleterious in its general effect. I believe the perfect articulation of the teeth is the true physiological basis towards which our operations should be directed. I have been criticised for assuming a perfect articulation as a standard, because I have not been able to show an absolutely perfect articulation; but this criticism is misleading and unjust.

In a paper on the "Dental Arches of Man" (read April 12, 1887, before the New York Odontological Society, and published in the

[1] The editor and publishers are not responsible for the views of authors of papers published in this department, nor for any claim to novelty, or otherwise, that may be made by them. No papers will be received for this department that have appeared in any other journal published in the country.

[2] Read before the American Dental Society of Europe, at Heidelberg, August, 1891.

*Dental Cosmos* in July of the same year) I showed the principles of perfect articulation, and illustrated the same not only by diagrams, but by models of mouths; of the latter, some were so perfect, except in minor details, as to well illustrate the perfect arch.

One may recognize that a defect exists without recognizing all that it involves. In order to realize the real import of an imperfection one must know what perfection is, how manifest, and what it implies, otherwise the attempt to correct faults will often result simply in changing one defect for another, perhaps a worse one.

We benefit our patients in proportion as we make their teeth approach the standard of perfection, and this cannot be done blindly and without a knowledge of what a correct articulation is and what it is able to accomplish, so it seems absurd to hear the statement "that we always have to do with imperfect articulations, and therefore the perfect standard is of no use to us."

That argument ought to be made use of only by those men whose vision is so short that they go cutting and digging about the mouth with perhaps the one noble idea of tooth salvation, but apparently regardless of *what the teeth were made for;* but theirs is not "*whole salvation*" which our Methodist friends would say "brings happiness as its fruit," but rather the miserable inheritance of unending repentance.

I have no patience for the dentists of the present day who go chopping about the mouth like the bushwhacker dentists before the glad day-dawn of dental science.

Nature never loses sight of perfection. *Deformities and useless variations are eternally destined to die out sooner or later*, and the fittest survive. In the slow process of evolution is one abiding faith.

The *standard* of perfection of an organ can never be lowered or diminished so long as its functional need exists.

While there are probably few perfect articulations, there are many that are imperfect; yet it is possible to learn what is perfect and what tends towards perfection, by observing articulations which most nearly answer the functional requirements; in time one comes to detect the discordant elements which, eliminated, leave a correct idea of perfect articulation.

In trying to discover that form of articulation most favorable to its greatest usefulness, permanence, and regularity, it became evident that some other method was needed than observation in the mouth, where only the outside of the teeth could be seen in contact, or with whole models from which only an imperfect idea of the inside striking of the teeth could be had.

To overcome some of these difficulties the method of vertically cutting the articulated models from front to rear, and with triple hinge permit to open laterally as well as vertically, was devised. Fig. 19 represents such a model, the interior view of the articulation being directly exposed to view.

Antagonism seems to express the prevalent idea of articulation; but antagonism is just what is most objectionable in an articulation.

Teeth antagonize when they strike only here and there and prevent the other surfaces from touching. Figs. 17, *Y* and *Z*, external and internal views, and Figs. 20, *a* exterior and *b* interior view, are good examples of an antagonizing articulation. When thus arranged they may punch the food, but they cannot chew it. On the other hand, a correct articulation of the teeth secures their very highest masticatory functions. In other words, a perfect articulation is the harmonious adjustment to each other of two most beautifully complicated, uneven, triturating surfaces in such a way as to permit all the movements of mastication, each prominence or depression having special reference to the normal movements of mastication, and to change their form or direction would be to render such movements impossible.

When thus perfectly arranged the masticating surfaces slide upon and into each other, constituting a self-sharpening machine, made up of a complicated system of inclines, so balanced and bound together as to be practically permanent.

The forces being thus widely distributed, wear goes on slowly on all the surfaces alike, and the physiological process of pulp protection is never, or at least very seldom, overtaken by exposure of sensitive fibrillæ, as is often the case when but little masticating surface can be employed.

I show you a model from the mouth of a man sixty-three years old (Figs. 21, *a* and *b*). Although defective in some respects, nature did her work so well that dentists have never had much chance to make it worse, for no teeth have required to be filled except some small cavities in the wisdom teeth, and that recently. I don't know when the absent wisdom tooth was lost. The points of the cusps are worn hardly any more than the other surfaces of the teeth, and yet it may be inferred that the gentleman used his teeth with some vigor, for he consulted me on account of having accidentally split longitudinally a perfectly sound bicuspid while eating.

In my former paper I was under the impression that perfectly articulating teeth would finally wear flat, owing to the shortening of the bite, and the under jaw coming forward, the ends of the incisors and the cusps thus disappearing coincidently; but this case illustrates how the perfect adaptation to its function may tend towards the preservation of an organ.

I do not wish to weary you with too many details of the articulation, but refer you to my former study and paper, "The Dental Arches of Man," already mentioned.

I will simply run hastily over some of the old drawings (for convenience the original numbering is here retained), together with a few others and several new models, dwelling particularly only upon such points as seem not to have been usually understood in that paper.

Figs. 1, *A*, *B*, *C*, and *D* are the drawings which, though not perfect, represent an approach at least to the typical arch. I will drop the word typical in this place, however, as some have objected to its application to them, and simply call these very good arches; arches which combine most of the desirable qualities of articulation, such as continuous grinding surfaces, good contact for the most part inside and out, extensive contact possible on many planes without interfering with individual prominences. Slight overshutting of superior incisors permitting the cutting movement without throwing the back teeth too far out of relation. The lower incisors capable of moving backward upon the posterior inclines of the upper incisors just sufficiently to conduct the cusps of the back teeth into their normal depressions with the same sliding contact; the rotary motion being permitted without the normal extent of contact being disturbed, either by too long cuspids or by such malposition of the teeth as to entangle the cusps, for it must be borne in mind that when a cusp by a false contact interferes, being out of relation with the rest of the surfaces, the movement is either arrested, or if continued, the effect of the rotation is destroyed because one cusp must raise over the other, and that separates the other surfaces.

To resume, we find the arches mutually supporting and tending neither to irregularity, contraction, spreading, separation, anterior projection, nor flattening; wear evenly distributed, and consequently slow.

Altogether sufficiently well combined to give a fair idea of a perfect arrangement, Figs. 22, *a* (exterior) and *b* (interior view), show another very good articulation.

Fig. 3 develops the idea of the relation of regularly uneven surfaces, and of progressive contact from one plane to another. I say regularly uneven surfaces, because the unevenness recurs at regular intervals.

Fig. 4 is the application of Fig. 3 to the arches of man, the dotted lines representing the lower jaw in the forward position, as during the act of biting or cutting.

Fig. 5 shows how a long canine might destroy the effect of rotation by separating the molars, or how too far overshutting incisors would, during the act of cutting, widely separate the molars.

Now I must tell you that diagrams Figs. 4 and 5 are correct in regard to the divisions of the teeth upon one vertical plane only, and here is the point to understand of the whole question. They represent the teeth as they appear from the outside, the points of the upper cusps being just opposite the space between the lower teeth.

This of itself alone would be an element of weakness, the teeth in the bicuspid region would tend to fall apart by such a mutual wedging action, and the molars, being left without support, would tend to disruption or splitting, an element, by the way, sometimes worth considering in treatment of weak, broken-down teeth.

In Fig. 8 the teeth have been separated and the interior teeth consequently forced forward by just this defective arrangement; and although we find a very good articulation in other respects, in this we see an essential defect, which prevents the permanence of the position of the teeth.

This defect often occurs after early extraction of the first molars, or premature extraction of the deciduous teeth, for the bicuspids straggle into false positions, their cusps falling into the spaces (which are sometimes held open permanently), and usually by their entanglement preventing the normal motions of the jaws. (See Fig. 23.)

It has just been stated that Fig. 4 correctly represents the articulation upon one vertical plane; this plane corresponds with the curved line 2 of Fig. 9.

To understand these curved vertical planes and Fig. 9, place a piece of card-board flatly between the teeth of an articulated model, letting it extend out beyond the teeth, then draw a line from the incisors to the third molar, just touching the outside of the upper teeth, and the resulting line will be line No. 1 of Fig. 9. Another line drawn in the same fashion, only touching the outside of the lower teeth, will give line No. 2 of Fig. 9. Prick through the card-board and the relation of these lines will at once be seen. The next line at the same distance, if the outside of the teeth were removed, would be line No. 3, which would pass through the bottom of the groove in the middle of the masticating surface of the upper molars and bicuspids, and through the outer cusps of lower molars and bicuspids and the cutting edges of lower incisors, as indicated in the diagram, and so on.

I have heretofore endeavored to show how the closed teeth should form one continuous self-supporting arch, "each jaw being," as Dr. Dwinelle once aptly remarked during the discussion of this subject, "a perfect matrix for the other;" yet in conversation with a number of eminent dentists since that paper appeared, I have not found one who grasped the idea of the cross or binding articulation of the teeth, especially the bicuspids, and the general impression is, that teeth ought to articulate as in Fig. 8, which has been already shown to be erroneous.

If I can make clear this one point of pure anatomy I shall feel that this paper has been of service to dentistry and mankind.

From the outside of any fairly well-arranged set of teeth, they can be seen as they appear in Fig. 4 (compare with Fig. 1, *C;* Fig. 21, *b;* Fig. 22, *a;* and Fig. 24, *a*); but look at it from the inside of a split model, and, if the articulation is good, the inner upper cusps do not shut into the spaces between the lower teeth and drive them apart, but instead, the cusp strikes into the depression of the next forward tooth, thus crossing over the space and binding the two lower teeth together. (See Fig. 1, *d*, and Fig. 22, *b*.)

If a vertical section were made on the line of the upper inner cusp, which is the same as line 4 of Fig. 9, the divisions of the teeth would appear as in Fig. 11, where we have the lower teeth bound together and prevented from being separated; but on this plane alone the tendency of the upper teeth would still be to separate, but another vertical section or view of the articulating line at 3 of Fig. 9—*i.e.*, through the points of the outer cusps of the lower teeth—would show the divisions of the teeth to be reversed (see Fig. 10), for here the upper teeth are permanently secured, while the lower teeth tend to separate.

It therefore follows that the two arches are mutually supporting, yet, without the split models it would hardly be suspected.

This crossing or binding of the articulation of the bicuspids (there is the same crossing in the molars) is most beautifully seen in the articulation of the teeth of anthropoid apes. (See Figs. 24, *a* and *b*.)

I have previously shown that the normally-arranged human teeth touch all around in both arches.

In order to be well articulated the teeth must be regularly placed and correctly inclined.

The most common irregularity of the teeth is the irregularity of the position of the masticating surfaces, and yet but little attention is given this matter in works upon irregularities, the attention being mostly confined to the deviations of the external curves or alignment of the teeth; yet if the former were attended to, the latter would necessarily be corrected, and more permanently so than is usually the case.

When teeth are regular and well articulated, they remain so because the forces and resistances are evenly balanced.

On the other hand, as the articulation is made up of a series of perfectly-balanced inclines, it follows that when anything removes one surface, whether an extraction, decay, operation, or badly-constructed regulating or other apparatus, undue force falls upon other inclines with the certain result of changing the position of the tooth or teeth.

I have hardly ever found what I could call a good articulation a few years after teeth had been extracted, and the same is true when the teeth have been cut away between, or when the cusps had been carelessly removed while finishing fillings in the grinding surfaces.

Such teeth antagonize but do not articulate, and cusps strike cusps, point to point (see Fig. 20), instead of passing between each other like cogs; and the motions of mastication are interfered with, especially the rotary, which is the essentially grinding motion, and hence the rapid wear of the limited number of antagonizing points goes on, as already spoken of.

Largely on account of bad articulation irregular teeth tend to become more irregular. Growth may improve some cases, but so far as a bad articulation goes it is always unfavorable to regularity of the teeth.

Much harm is done by the use of regulating appliances which change the articulation without improving it, and it is almost a universal fact that unless an improvement can be made in an articulation there will be no permanent improvement of the irregularity.

Finally, the articulation is the only permanent retainer to be depended upon.

The teeth will move till they find the best contact that circumstances can offer, and that movement often never ceases, because the forces never find equilibrium.

Before disturbing the articulation of a fixed irregularity, it is best to consider whether such disturbance can be overcome, and the articulation again made as good; if not, the chances are that the result will be worse than the original condition, and for the ultimate result we must wait not only "till the teeth become firm," as we say, but until they cease to move.

There is much yet to learn in regard to the meaning of the elevations, depressions, overshutting, shelving, interlocking, binding, curves, and inclines of the articulation, in their relation to biting, cutting, tearing, crushing, and grinding movements of mastication.

In the treatment of our patients it is hoped that if we cannot all see our way clearly upon this matter, that we may at least see far enough not to make the articulations worse by our operations than they are when brought to us.

I will renew my endorsement, made before the International Dental Congress at Paris, of the use of Dr. Bonwill's articulator for the arrangement of artificial teeth. At the same time I also called attention to a paper by Mr. F. H. Balkwill, L.D.S., R.C.S., of Plymouth, England, but as the latter remarks have not appeared in any of the published reports, I again call attention to his remarkable paper, "On the Best Form and Arrangement of Artificial Teeth for Mastication," read in 1866, and published in 1868 by the Odontological Society of Great Britain, but only brought to my notice since the publication of my paper of 1887, before referred to.

## Figures and Tables

**Fig. 1 A. f1:**
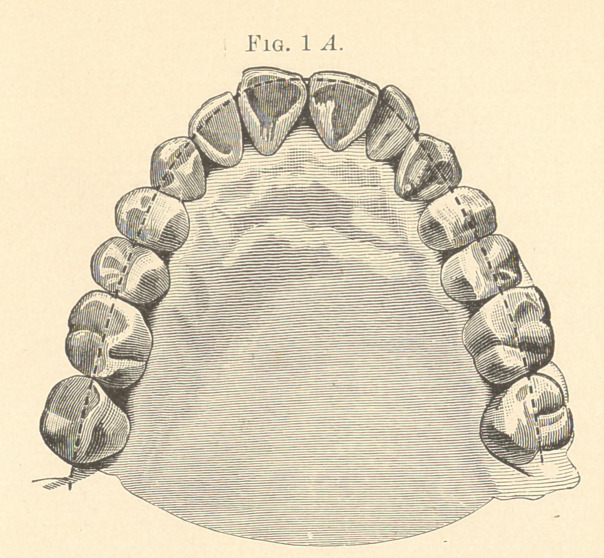


**Fig. 1 B. f2:**
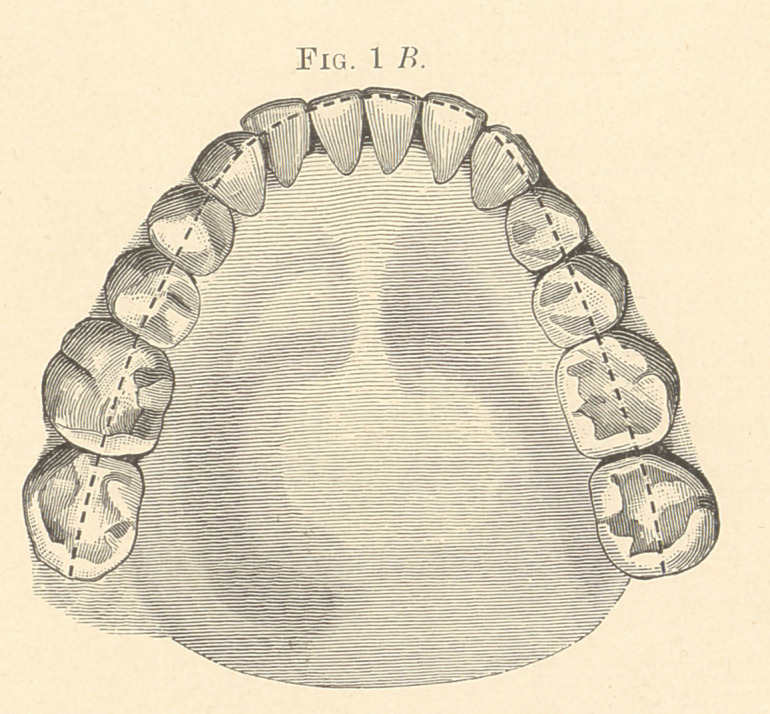


**Fig. 1 C. f3:**
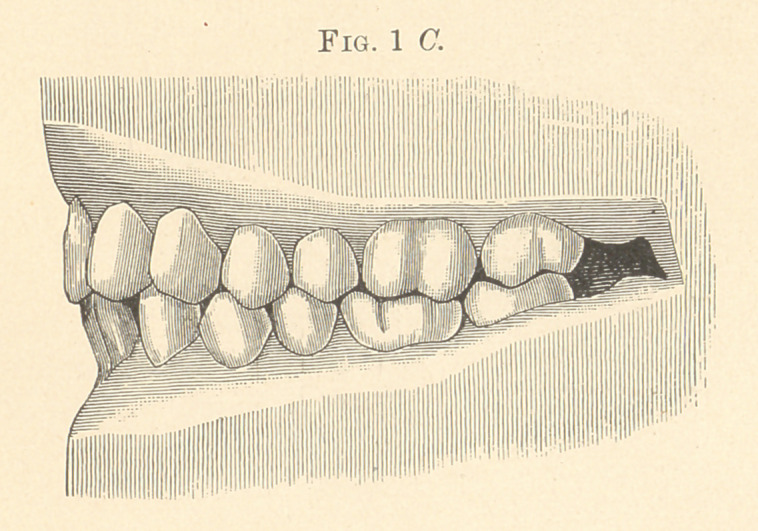


**Fig. 1 D. f4:**
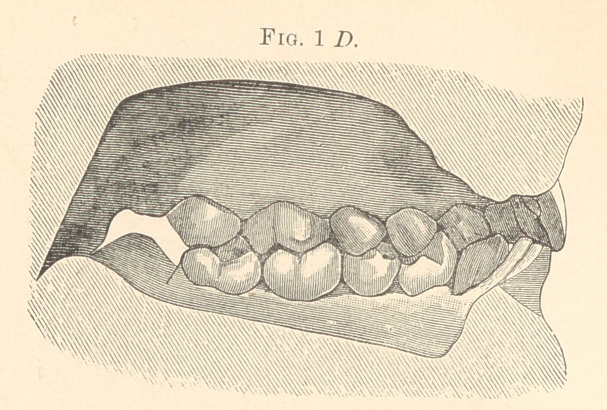


**Fig. 8. f5:**
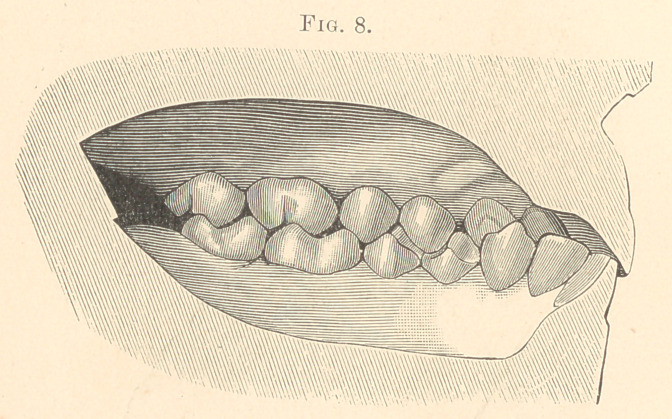


**Fig. 9. f6:**
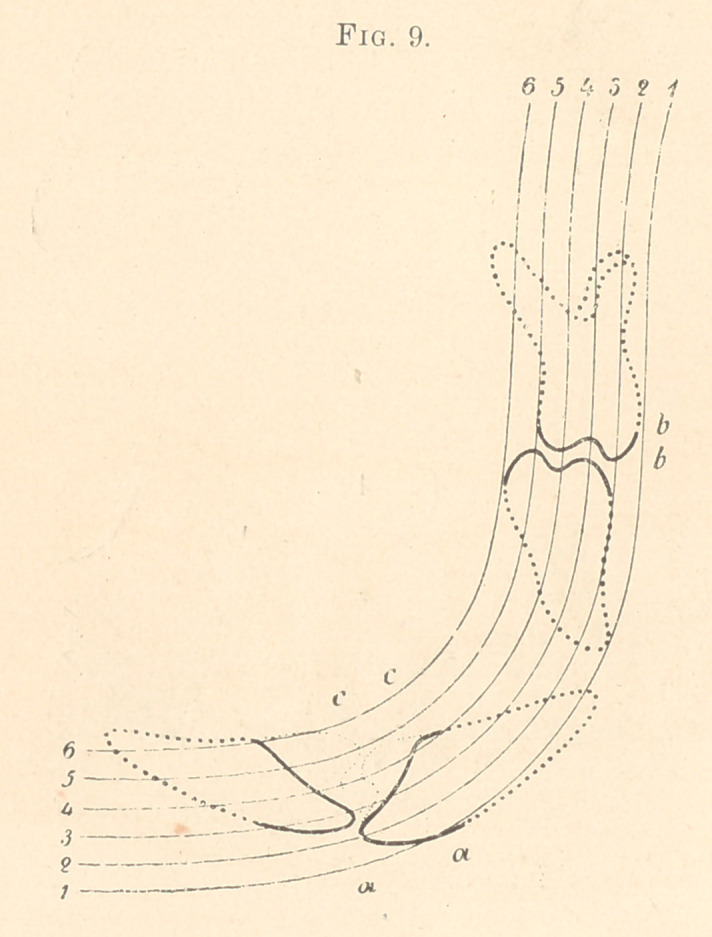


**Fig. 3. f7:**
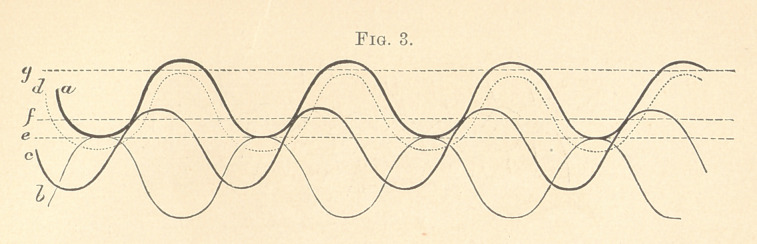


**Fig. 19. f8:**
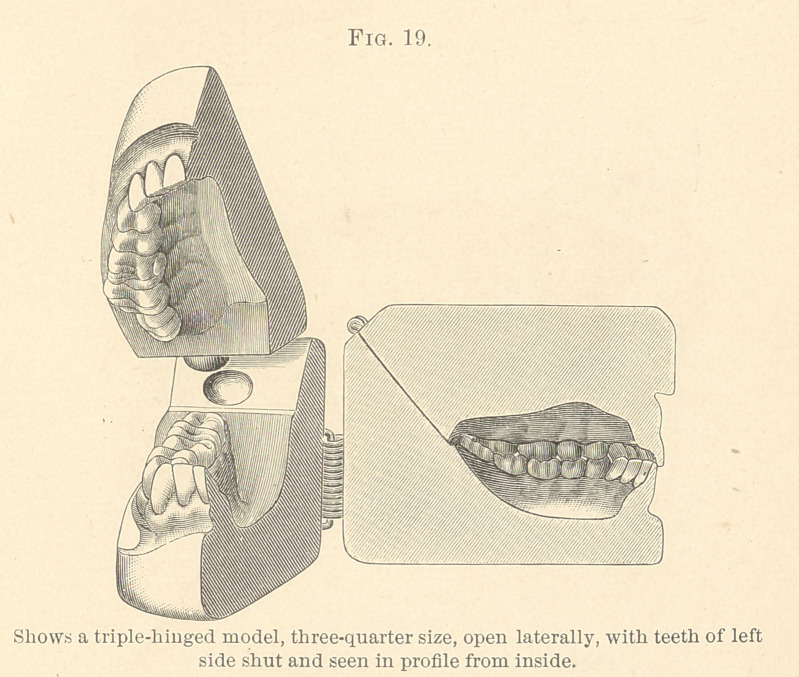


**Fig. 23. f9:**
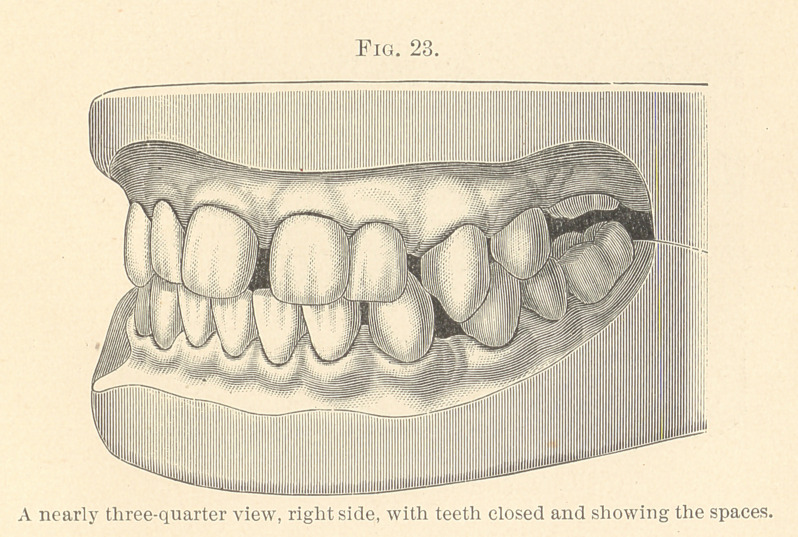


**Fig. 4. f10:**
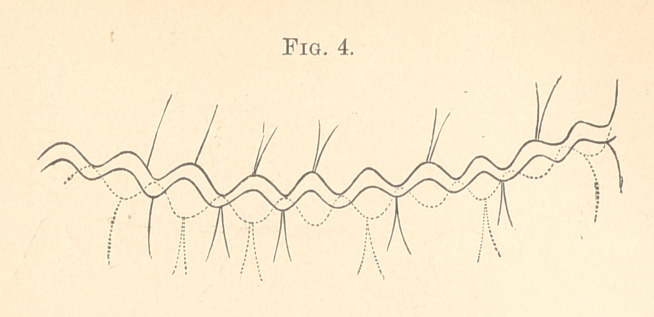


**Fig. 20 a. f11:**
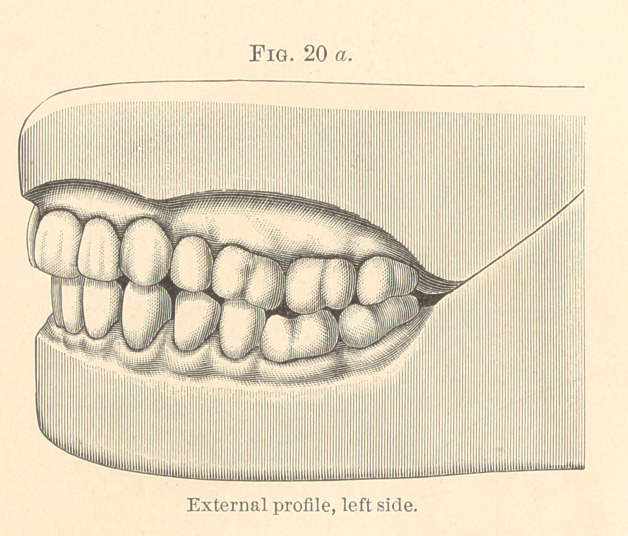


**Fig. 20 b. f12:**
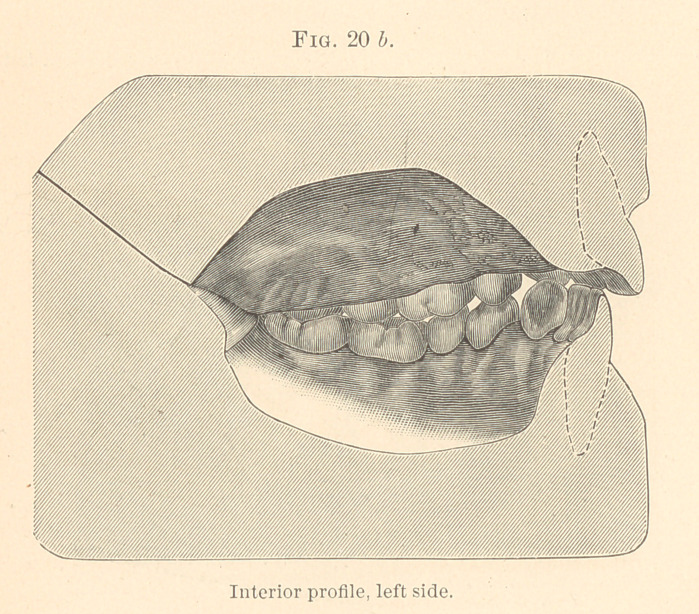


**Fig. 5. f13:**
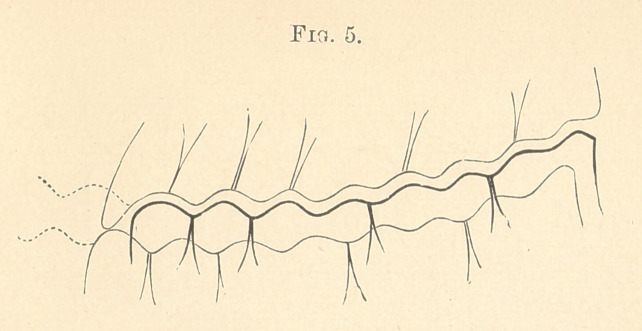


**Fig. 21 a. f14:**
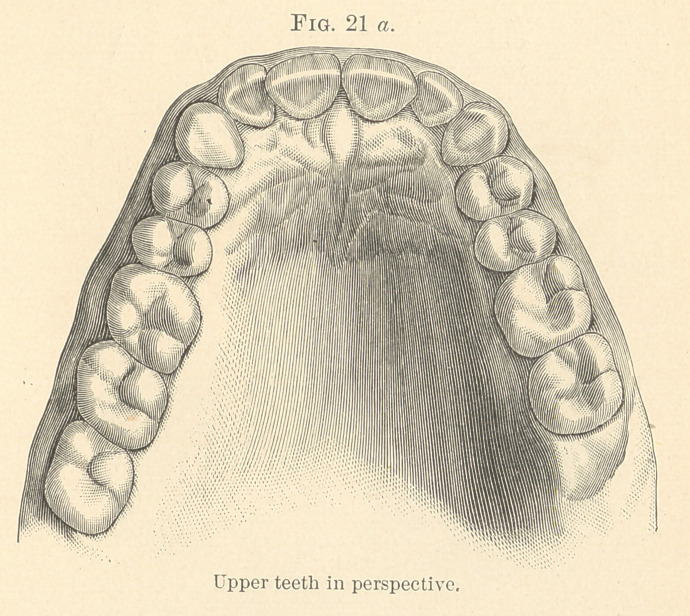


**Fig. 21 b. f15:**
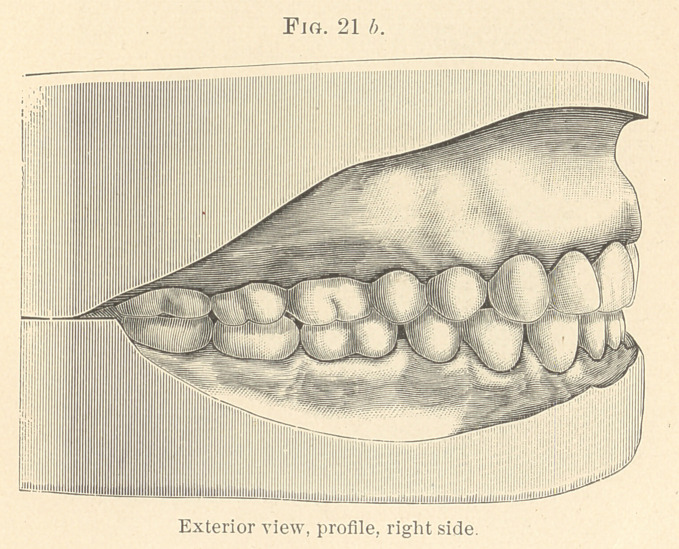


**Fig. 10. f16:**
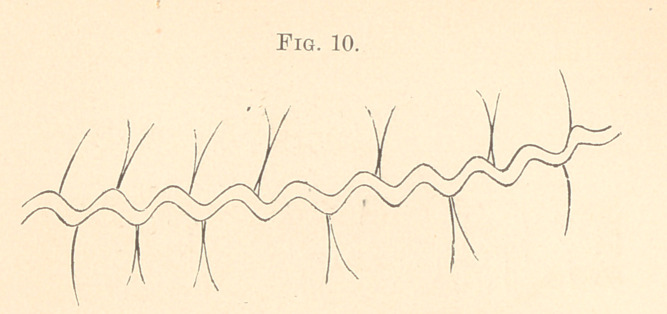


**Fig. 11. f17:**
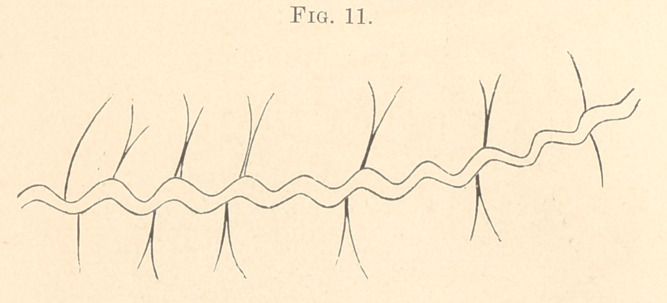


**Fig. 17 Y. f18:**
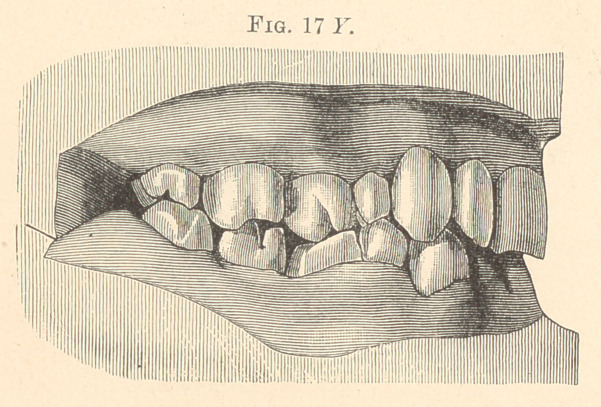


**Fig. 17 Z. f19:**
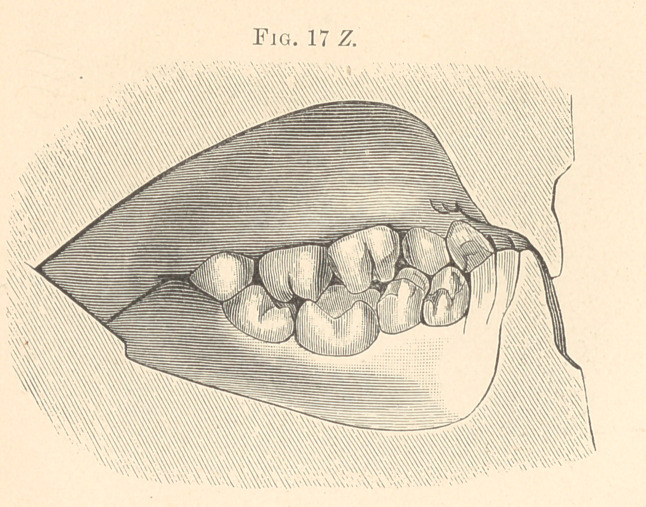


**Fig. 22 a. f20:**
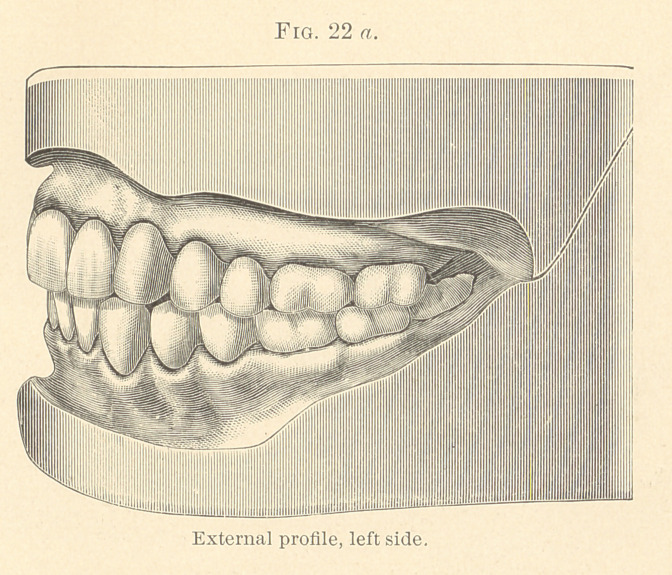


**Fig. 22 b. f21:**
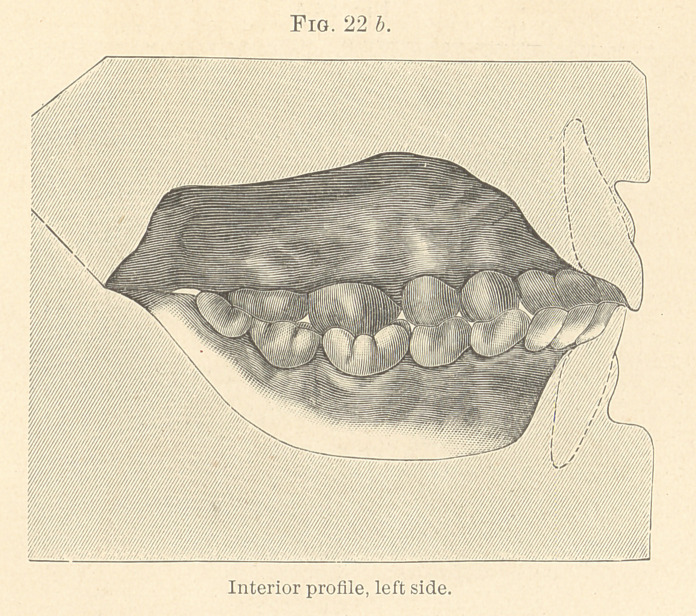


**Fig. 24 a. f22:**
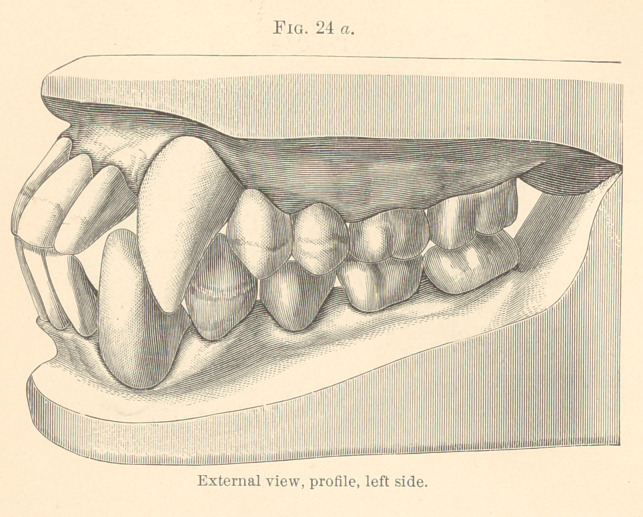


**Fig. 24 b. f23:**